# Ohio Dental College Association

**Published:** 1866-03

**Authors:** 


### OHIO DENTAL COLLEGE ASSOCIATION.

This association met on the the 20th of February with a very full representation of its membership.

Much important business was transacted, for a detailed account of which, see minutes published in the Register. The most lively interest was manifested by the association, and the prosperity of the school seemed to infuse new zeal into the members, so that they pledged far more effort for the future, than has ever been put forth in the past, and we doubt not the pledges will be fully redeemed. Among the more important operations of the society, was the election under the amended charter, of a new board of Trustees.

According to the charter, they must all be members of the association; and they are all active members of the profession, and men too, having the good of the profession, and especially this Institution most earnestly at heart. The members of the former board were nearly all outside of the profession, and could not be expected to have the same lively interest in its welfare, as those engaged in its active duties; but notwithstanding that such was their position, the profession owes them a debt of gratitude that will forever remain unpaid except in good wishes and kind remembrances.

They bore the College through difficulties and trials, that in the hands of a less energetic Board, would have involved it in ruin. Let us then with never failing gratitude remember our retiring Board of Trustees.

The new Board, consists of the following persons, viz: Dr. James Taylor, Cincinnati Prest. H. A. Smith, Cincinnati, Sect. Dr. A. Berry, Cincinati. Dr. B. D. Wheeler, Cincinnati. Dr. W. W. Allport, Chicago Ill. Dr. H. J. McKellops, St. Louis, Mo. Dr. Geo. W. Keely, Oxford, O. Dr. W. H. Margan Nashville, Tenn. Dr. A. S. Talbert, Lexington, Ky. They immediately organized and went to work arranging such plans and details as will tend greatly to the prosperity of the school. The association appointed Committies at various places throughout the West, to receive any contributions that may be made to the College in any form, such as specimens of any kind that may serve as illustrations, chemical apparatus, appliances and fixtures for the practical departments; *or material aid.* The Committees are as follows,

Dr. A Berry, of Cincinnati, CHAIRMAN.
" W. H. Morgan, of Nashville, Tenn.
" G. W. Redman, of Louisville Ky.
" A. S. Talbert, of Lexington, Ky.
" M. DeCamp, of Mansfield, Ohio.
" J. B. Dunlevy, of Pittsburg Pa.
" H. J. McKellops. St. Louis, Mo.
" A. M. Leslie, " "
" Geo. H. Cushing, Chicago Ill.
" A. A. Blount, Springfield, Ohio.
" P. G. C. Hunt, Indianapolis, Ind.
" W. P. Horton, Cleveland, O.

These will all doubtless respond most fully to the expectations of the association in the performance of the work assigned to their charge.

